# Exploring the dynamics of the quality of HIV care experienced by female sex workers living in the Dominican Republic

**DOI:** 10.1371/journal.pgph.0001479

**Published:** 2023-04-28

**Authors:** Tahilin Sanchez Karver, Clare Barrington, Yeycy Donastorg, Martha Perez, Hoisex Gomez, Kathleen R. Page, David D. Celentano, Katherine Clegg Smith, Deanna Kerrigan

**Affiliations:** 1 Department of Health, Behavior and Society, Bloomberg School of Public Health, Johns Hopkins University, Baltimore, Maryland, United States of America; 2 Department of Health Behavior, Gillings School of Global Public Health, University of North Carolina, Chapel Hill, North Carolina, United States of America; 3 Instituto Dermatológico y Cirugía de la Piel “Dr. Huberto Bogaert Díaz”, HIV Vaccine Trials Research Unit, Santo Domingo, Dominican Republic; 4 Division of Infectious Diseases, Johns Hopkins University School of Medicine, Baltimore, Maryland, United States of America; 5 Department of Epidemiology, Bloomberg School of Public Health, Johns Hopkins University, Baltimore, Maryland, United States of America; 6 Department of Prevention and Community Health, Milken Institute School of Public Health, George Washington University, Washington, District of Columbia, United States of America; University of Canberra, AUSTRALIA

## Abstract

Despite increased attention and efforts to improve HIV care among female sex workers (FSWs), they continue to have suboptimal HIV outcomes. Exploring the socio-structural dynamics related to the quality of HIV care received by FSWs is critical to further strengthen interventions to improve their HIV care continuum outcomes. In this study, we conducted two rounds of qualitative in-depth interviews with 20 FSWs living with HIV in the Dominican Republic to explore how healthcare experiences contributed to their quality of HIV care. Data was analyzed using a thematic analytic approach exploring diverse structural and relational aspects of the quality of HIV care affecting FSWs as they navigate the clinic environment. Results indicated that quality of HIV care was influenced by both structural and relational factors within clinics. At the structural level, insufficient stock of antiretroviral therapy and the financial burden created by HIV care related costs hindered FSWs’ satisfaction with their current HIV care and presented a barrier in FSWs’ ability to access HIV care services. Quality of care was also closely linked to relational aspects of the HIV care environment, including FSWs’ relationship and communication with their clinical providers, as FSWs often expressed their satisfaction with HIV care experiences based on these interpersonal factors. Lastly, personal agency emerged as an important factor contributing to the quality of HIV care, specifically as FSWs’ treatment literacy resulted in greater advocacy and demands for quality care. Programmatic efforts should be directed to improving the quality of HIV care experiences of FSWs in the clinic environment. These include addressing resource shortages, promoting positive and effective patient-provider relationships, and facilitating HIV treatment education opportunities for FSWs.

## Introduction

Globally, female sex workers (FSWs) are a multiply stigmatized population experiencing widespread socio-structural constraints contributing to their disproportionate burden of HIV [1–11]. Global HIV prevalence among this group is high at 10.4%, with the Latin America and Caribbean region having an estimated prevalence of 4.2% among FSWs [6]. Across all settings, FSWs have been found to experience significantly higher levels of HIV compared to all women of reproductive age between 15 to 49 years [1,2,6,12–17]. The evidence suggests that FSWs between 15 and 49 years have 13.5 times greater odds of being HIV-infected compared to women overall in low- and middle-income countries [12,18]. In the Dominican Republic (DR), there is an increased number of women in sex work, raging between 60,000 to 100,000, working in diverse environments such as venue (e.g., discos, bars, and clubs) and non-venue based settings (e.g., street, cell-phones) [9,18]. Similar to global trends, in the DR, FSWs experience a disproportionate burden of HIV having nearly 5 times greater odds to be living with HIV compared to other adults (HIV prevalence of 4.4% [9] vs. 0.9% [19]).

Despite increased efforts to improve HIV treatment and care programming for FSWs [20], they continue to have suboptimal HIV outcomes [6,21,22]. For example, a systematic review found that among FSWs living with HIV, only 38% are currently on ART and 57% of FSWs on ART are virally suppressed [23]. Some examples of socio-structural factors contributing to FSWs’ poor HIV outcomes include experiences of stigma and discrimination, violence, general low literacy, and poverty and social instability [7,20,24,25]. In particular, in the DR, despite sex work not being subjected to punitive regulations or to criminalization [19,26], FSWs experience significant stigma and discrimination often leading to fear of authorities, and are not afforded the same protection rights as other women [7].

In the healthcare environment, studies have found key factors that contribute to the poor attainment of HIV outcomes, such as linking to HIV care, ART initiation and discontinuation, at both the provider and clinic levels [20,24]. At the provider level, research has highlighted the importance of the patient-provider relationship, with poor treatment from healthcare providers being linked with obstructing positive experiences in HIV care environments [20,24,27–29]. For example, stigma related to sex work inhibits FSWs’ ability to speak about their occupation candidly with healthcare professionals and as a result may stop them from obtaining the comprehensive healthcare they need, including violence counseling and treatment for HIV and sexually transmitted infections (STI) [27–29]. In addition, evidence suggests that healthcare providers exhibit greater bias towards FSWs in general compared to those individuals living with HIV not engaging in sex work [27]. In fact, there is an independent (negative) association between sex work stigma and access to healthcare services [3,27,30]. One study found that healthcare workers engage in discrimination against sex workers at a high rate, with nearly 72% of FSWs in the study experiencing some form of sex work related stigma by healthcare providers [31]. This finding is alarming as it could effectively lead to the avoidance of formal health services among this group [31,32]. Furthermore, poor treatment by healthcare providers has also been associated with decreased ART initiation, increased ART interruption, and declines in ART adherence [24,32,33]. The clinic environment also shapes the experiences of FSWs as it relates to their HIV care and could inhibit positive HIV outcomes among this population. Clinic-level factors such as costs associated with HIV care, distance to healthcare facilities, rigid clinic policies, and healthcare facilities’ inability to meet the demands for ART, have been found to obstruct HIV treatment and care [20,24].

Despite existing evidence on clinic and provider factors facilitating or inhibiting optimal HIV outcomes among FSWs, research exploring the quality of HIV care among this population has been limited. More evidence is needed to explore how the clinical context and provider dynamics influence quality of HIV care among FSWs. This study aimed to explore this relationship among HIV positive FSWs living in the DR.

### Theoretical framework for understanding quality of HIV care

In this study, our conceptualization for quality of HIV care was influenced by Donabedian’s quality assurance in health care framework. Under this framework, Donabedian acknowledges that quality of care is a “remarkable difficult notion to define” given the diverse attributes in the care process that are important when it comes to assessing the quality of care received and its effect on society at large [34]. Despite this acknowledgement, under Donabedian’s framework there is an understanding that *structure* and *process* of care elements in the care pathway affect the quality of care of individuals, and their ability to attain a desired health outcome [35]. *Structures of care* is defined as the conditions under which care is provided to patients and could include aspects more directly linked to the health care structure, such as cost of care [35]. Meanwhile, *process of care* is defined as the activities that constitute receiving that care including *relational* aspects such as treatment by healthcare providers [35]. We used Donabedian’s framework to guide our exploration of the care experience of FSWs living with HIV in the DR, and how domains related to the structural and relational aspects of care influenced their quality of HIV care.

## Methods

### Study design

The current study was embedded within the NIMH-funded parent study titled “Stigma, cohesion and HIV outcomes among vulnerable women across epidemic settings” (R01MH110158). The parent study was a longitudinal observational cohort study conducted in Iringa, Tanzania (n = 208) and Santo Domingo, DR (n = 201) from 2016 to 2022 and assessed the role of socio-structural and behavioral factors along the pathway to viral suppression among FSWs living with HIV. In the DR, women were followed prospectively at 0, 12 and 24 months, and eligibility criteria included being 18 years or older, with a confirmed HIV-positive diagnosis and having reported exchanging sex for money in the last month prior to enrollment. Participants in the DR were largely recruited from an already established cohort, *Abriendo Puertas* (Opening Doors) [8,9], and recruitment was enhanced through the aid of peer navigators, key informants, and participants themselves.

As part of the parent study, a subsample of 40 women (n = 20 in Tanzania and n = 20 in the DR) from the original cohort were invited to form part of a qualitative longitudinal cohort study aiming to explore the dynamic context of social cohesion and HIV and sex work related stigmas among FSWs in relation to HIV services and outcomes. This qualitative longitudinal cohort study comprised of three separate rounds of data collection and was ongoing during the analysis of the current study. The current study is based on the qualitative data from the first two rounds of semi-structured, in-depth interviews (IDIs) conducted with the qualitative subsample from the DR.

### Study participants and data collection

Participants for the qualitative subsample were recruited using a stratified purposeful sampling approach [36] based on viral suppression at baseline which resulted in a cohort of 20 participants, 10 virally suppressed (viral load less than 400 copies/mL) and 10 not virally suppressed (viral load 400 or more copies/mL). Viral suppression was assessed through biological data collected by our local research team at the *Instituto Dermatológico y Cirugía de Piel “Dr*. *Huberto Bogaert Díaz”* (IDCP) during baseline (at 0 months). During the baseline visit, all participants forming part of the parent study consented to participate in a 10 mL blood draw to assess their viral load. Viral load was assessed using polymerase chain reaction (PCR) technology with the Roche Amplicor HIV-1 Monitor Test. Women in our study reported receiving HIV care from both public and private comprehensive HIV care facilities in the DR.

All IDIs were audio-recorded and conducted in Spanish by two trained IDCP Dominican female qualitative researchers (with postgraduate degrees (MS)) in a private office at the IDCP after securing informed consent from all participants. All interviews were conducted one-on-one with the participant and a qualitative researcher. Female qualitative researchers in this study have had extensive engagement with the female sex worker community in the DR, thus allowing for more meaningful engagement with participants given their ability to build rapport and trust with participants during the interview process. The ability of our qualitative researchers to understanding the ethos and socio-cultural dynamics of the sex worker community in the DR allowed for more open and candid dialogues increasing the rigor and trustworthiness of the data [37]. All 20 participants included in this study completed the first two rounds of semi-structured, IDIs. The first round was completed between October and November 2018, whereas the second round between December 2019 and February 2020.

The semi-structured IDI guides for both rounds of interviews contained a combination of open-ended questions and probes exploring participants’ perspectives and opinions related to their current health status and practices use to take care of their health; how participants manage their HIV status; participants’ ability to access HIV care services and experiences with these services; and participants’ ability to adhere to ART medication regimens. The focus of the current study centered around women’s experience with HIV care services. Sample questions used in the IDI guides include: Can you talk to me about the process of starting to receive HIV care and treatment after your diagnosis? Coming back to the present/today, how are you doing managing your HIV care and treatment? What information does your doctor (or other health professional) give you to help you know how you are doing with your HIV management? Does your HIV doctor know about your work as a sex worker? Can you tell me more about this? Each IDI lasted approximately 60 minutes.

### Data management and analysis

All interviews were labelled with unique identifiers, transcribed by in-country staff from the IDCP with expertise in audio transcriptions, and analyzed in Spanish by an independent coder with training in qualitative data analysis, experience working with the female sex worker community in the DR and native Spanish speaker. Select quotes were translated to English for the purpose of manuscript development. Data analysis focused on topics surrounding experiences with HIV care, such as: (1) Perceptions of their current HIV care; (2) Understanding of their current HIV treatment; (3) Facilitators and Barriers to receiving HIV treatment and care; and (4) Recommendations to improve their HIV treatment and care experiences. We began our qualitative analysis by reading each transcribed interview thoroughly and writing an analytic summary for each participant’s interview summarizing their HIV care experience. These analytic summaries allowed us to identify recurring and salient themes in the data. Next, together with their analytic summary, each transcription was read again prior to coding to allow for a richer immersion into the contextual realities of each participant. We then proceeded to analyze interviews using a thematic analysis approach [38] applying a combination of both inductive and deductive coding. The two rounds of interviews per participant were analyzed utilizing a repeated approach rather than exploring the interview data longitudinally [39]. As such, all data for each participant was used to explore their experiences in the HIV care environment to investigate their quality of care in-depth, but not necessarily looking at changes over time [39].

We developed a codebook using a set of *a priori* codes based on the IDI guide, study objectives, narrative summaries, and informed by our exploration into the structural and relational factors influencing the HIV care experience based on Donabedian’s framework for health care quality [35]. Additional codes relevant to the care experience emerged and were also explored. Throughout the analytic process, the coder engaged in routine notetaking utilizing memos to synthesize the data, allow for a mechanism to present the data to in-country qualitative researchers, and capture any relevant thoughts that could have implications in the conceptualization of results [40]. Furthermore, during the iterative coding process, saturation of themes was closely monitored to ensure that little to no new information was emerging from participants’ transcripts. Memos were reviewed, code outputs were synthesized across domains, and salient themes were analyzed. Furthermore, findings for each theme were systematically organized and presented during research debrief meetings with the study team, including the independent coder and qualitative researchers who conducted the interviews. These discussions allowed for the assessment of the dependability and confirmability of the results through engagement in detailed discussions to explore all of the results [41]. The Consolidated Criteria for Reporting Qualitative Research (COREQ) was utilized to aid the process of documentation and reporting [42]. ATLAS.ti version 8.4.4 [43] was used to manage the qualitative data.

### Ethical considerations

This study received human subjects research approvals from the Institutional Review Boards of the Johns Hopkins University Bloomberg School of Public Health (Baltimore, MD), and from *Instituto Dermatológico y Cirugía de Piel “Dr*. *Huberto Bogaert Díaz”* (IDCP) *Unidad de Vacunas e Investigación* and the *Consejo Nacional de Bioética en Salud* (CONABIOS) (Santo Domingo, DR). All participants provided oral informed consent prior to any study activity. Through this process, participants were informed about the study objective, rationale for conducting the study, study methods and protocols, and respond to questions or comments by the participants on study participation. Furthermore, in accordance with community practices in the DR, all participants received 400 Dominican pesos (approximately $7 USD) in compensation for their time and participation per interview.

## Results

As shown in **Table 1**, the median age of women was 40 years (range: 21–53 years). Most women were single, separated or widowed (70%), and had an educational attainment of primary school (55%). All women included in the sample were mothers. Regarding their HIV characteristics, the median number of years women had been living with HIV was 12 (range: 3–38), while the median number of years women reported being on ART was 9 (range: 2–22). The majority of women (85%) were currently taking ART, with most women on ART reported being adherent to their treatment (88.2%).

**Table 1:** Women’s sociodemographic and HIV characteristics (n = 20).

		n	%
***Individual Characteristics***			
Age (median, range)		40	(21, 53)
Education			
	Primary	11	55.0
	Secondary	7	35.0
	University or Post-Graduate	2	10.0
Marital Status			
	Single/never married	5	25.0
	Domestic union	6	30.0
	Separated	7	35.0
	Widowed	2	10.0
Number of children			
	1 child	4	20.0
	2 children	9	45.0
	3 children	7	35.0
***HIV Characteristics***			
Years living with HIV (median, range)		12	(3, 38)
Currently on ART			
	No	3	15.0
	Yes	17	85.0
Years on ART^		9	(2, 22)
ART Adherence in the last 4 days			
	No	2	11.8
	Yes	15	88.2

^n = 18.

**Fig 1:**
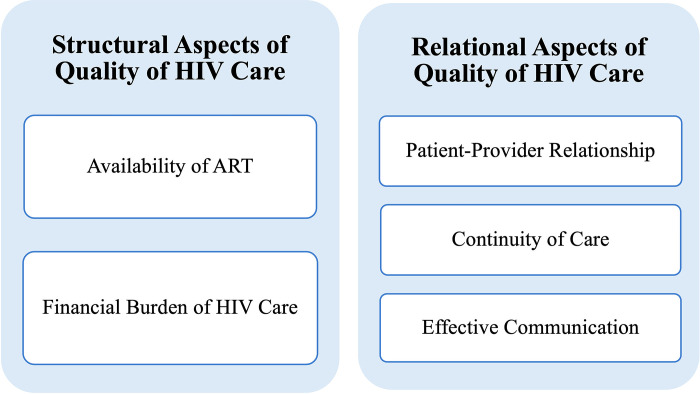
Salient themes related to the context and dynamics of quality of HIV care organized by structural and relational domains.

Results are organized into two domains: structural and relational aspects of quality of HIV care experienced by FSWs. Salient structural aspects relevant to the experience of quality of HIV care (**Fig 1**) included availability of ART and cost of care, while relational aspects influencing the quality of HIV care ***experience*** included the patient-provider relationship, continuity of care, and effective communication.

### Structural aspects of quality of HIV care

#### Insufficient ART stock in clinics

When discussing their HIV care experience, some women emphasized their frustration and anger with their clinics’ inability to provide ART to patients in a timely manner, often affecting their own ART adherence. In one instance, a participant described her experience going back and forth to the clinic to collect her ART, only to be turned around due to insufficient ART stock:

*“I worry*, *and right away I go back [to the clinic] and [the healthcare providers] tell me*, *come back tomorrow*, *come back the day after*, *that there are no [ART] medications and I tell him ‘you are going to let me die because I cannot last three days*, *four days without those medication*, *since I stop taking those [ART] medicines I feel like my body is like a*, *like a current that passes me*, *no*,*’… and I told him let’s see what [providers] are going to do*, *let’s see*, *because I can’t stand it [her side effects of not having her ART medication]*…*”* [32 years old, 7 years living with HIV]

For some of the women, these shortages even led to a change in ART regimen. In this next example, a woman explained her exasperation towards her doctor and her clinic because of their reluctance to provide her with her preferred ART regimen, which had worked well for her, due to insufficient ART stock. She recalled her provider trying to place her back on a previous ART regimen that had caused many side effects:

*“I told [my doctor]*, *I am not going to take the medicine*, *I am not going to take the Stocrin*, *I am not going to take that*. *Here [at the clinic] there are too many medicines that you can change me to*, *but ah there is no [ART medicines]*, *there was none and the shortage began*. *There are no [ART] medications so they gave me one and [then] a month later*, *it was scarce…I took a single month of that [ART medication] and already [you are telling] me [that there is not enough]*.*”* [53 years old, 38 years living with HIV]

Even among women who had not been affected by the ART stock shortage, there was still a fear that the ART shortage could impact their treatment. One woman commented the following:

*“[I am still with] the same [clinic]*, *with the same [ART] treatment*. *Though recently I was afraid… because there was a shortage of medicine [at the clinic]*, *I don’t know if you remember*, *there was a month that there was a shortage of medicine and there were people who were changed [to a different ART regimen]*. *There were people who took a medicine*, *then when they went to the [clinic]*, *there was no medicine and they were put on another one*, *and when the next appointment came*, *there was no [more medicine] and then they were put [on yet] another and I was stressed with that because I didn’t want to change my [ART medication]…Because my medicine works for me*.*”* [41 years old, 3 years living with HIV]

Not surprisingly, a recommendation to improve the quality of HIV care centered on the clinics’ ability to secure the adequate amount of ART stock in order to reduce the number of changes in ART regimen that some women have experienced, as expressed below:

*“Take good care of patients that live with [HIV]…because the lack of medicine is a problem*, *and my medications change a lot*. *I do not like that*.*”* [32 years old, 7 years living with HIV]

#### The financial burden of HIV care

Some women described the financial hardships that they experienced as a result of accessing HIV care. High clinic visitation frequency, mainly due to ART distribution (having to come more regularly to the clinics to pick up ART due to its limited availability), placed a significant financial strain on many women, as going to the clinics often implied being there for many hours. Some women could not afford to purchase food to eat while they wait to be seen by a doctor or to retrieve their medication. Some also explained that transportation alone, getting to and from their clinics, created a heavy financial burden. In the following quote, one woman explained the financial strain that is placed by transportation:

*“… from [the neighborhood where I live] I pay 45 [Dominican Pesos] to get on the bus*, *but sometimes the bus takes a long time and you have to leave early because at 12pm they close [the clinic] and you have to take a motorcycle for 50 Pesos…and then another 25 [Pesos] for a bus or 25 [Pesos] for a car*, *and that is already 75 [Pesos] and if you are going to pay 25 Pesos more to the clinic [for fees] well that is already 200 [Pesos*, *roundtrip]*, *now you don’t even have for breakfast*.*”* [53 years old, 20 years living with HIV]

The same participant also explained that given the financial strain caused by transportation, she is sometimes able to arrange for her provider to deliver her medication to her home or to collect her ART refill at another meeting point outside the clinic. She reported:

*“Yes*, *I call my [provider]*, *‘[hey*, *look*, *I don’t have [ART] medicine’ and sometimes she says ‘I’m going to take it to you’ or if not*, *’we are going to meet at [a local park closer to my home] because she [lives closer to me] and sometimes you don’t have the money to go to the clinic because it is 200 [Dominican Pesos] for the trip*, *and sometimes you don’t have it*.*”* [53 years old, 20 years living with HIV]

For some women not having enough money to pay for transportation led to missed HIV care appointments. As explained by one 41-year-old woman who has been living with HIV for 12 years: *“in the past I had difficulties*, *I would miss my appointments because I couldn’t pay the fare*.*”*

A few women described programs offered by the clinics that provided support for their groceries alleviating the financial hardship they are experiencing, which was exacerbated by costs associated with their HIV care. Women who had access to this type of program in the past spoke about it with nostalgia and with a desire to have the program operate regularly, as this is a type of help that appears to be unpredictable—they could only count on it some of the time. One participant recalled this service:

*“[In this clinic] they help me more because here twice I got my money for groceries*, *but then they said that they took it away*, *I don’t know why*, *but that money for groceries helped me a lot at home*.*”* [25 years old, 3 years living with HIV]

Lastly, despite the DR offering ART free of charge for people living with HIV, fear associated with the possibility of having to pay for the medication remains a stressor. One participant alluded to this by expressing fear of having the medication being offered at a cost. This 21-year-old woman, who was diagnosed at birth with HIV, explained that she has a child to take care of, and he comes first and *“no one should be paying for [ART] medication*.*”* This participant has also had difficulties picking up her ART given the financial struggles that she is experiencing, which are only aggravated by a clinic fee she would be responsible for when she goes to her appointments.

### Relational aspects of care

#### The patient-provider relationship

Women often recalled their own patient-provider relationships and their last interaction with their providers to offer insight into how they were doing and feeling about their HIV care. For many participants, being treated with respect and dignity by their healthcare providers was an indication of experiencing good HIV care. A 53-year-old participant who has been living with HIV for 38 years explained how she feels good about her current HIV care because her doctor treats her well, *“like family*.*”* In explaining how her current care was going, another participant expressed the following:

*“[Providers] treat me well [at the clinic] all the time*, *I don’t think there are people who do not go there because those people treat you well*, *it can be your final stage*, *[and they treat you] with a lot of love*, *with a lot of affection*.*”* [53 years old, 20 years living with HIV]

For some women the discussion around their relationships with providers and the type of treatment they received also involved the type of information that they were receiving from their providers. Women would expand on their discussion related to their views on HIV care beyond sharing personal accounts about their treatment by providers to how the information they were receiving was framed and delivered by their doctors (particularly in terms of the information needed to remain healthy). One participant explained:

*“[I feel] good [with my HIV care]*, *because they explain to you how you have to take care of yourself…they tell you that the main thing is [taking] your medications each day*, *that you should not miss your appointment*, *because sometimes you go to the appointment to look for the medications but you may have another problem that does not have to do with your medications*, *and [they also tell you] what needs to be tested*, *viral load*, *hepatitis B…”* [39 years old, 14 years living with HIV]

Furthermore, for some women having access to their providers outside of their regular clinic appointments to answer questions or concerns was also an attribute of a positive patient-provider dynamic. As one woman recalled:

*“[My providers] make things easier for me*, *as I am so far away*,*…I just have to write on WhatsApp…And I feel good because despite everything they try to help you as much as they can*.*”* [37 years old, 12 years living with HIV]

Women also focused on the type of emotional support that their providers gave them as part of a positive patient-provider relationship. Women would recall how their providers have asked them “how they are feeling?”, “how everything is going for them?” and these are important questions that showed them the type of caring practitioners they are and the type of support they could receive from them. One woman recalled the conversation she had with her provider when she was not managing her HIV diagnosis and treatment well:

*“[My doctor] spoke very well to me*, *she advised me about my life*, *how I have to live life*, *’don’t let people get to you*,*’ when I hear people talking about anything [about me]*. *She always thinks that I am very important*, *[more] than anything that ‘I have to love myself*.*’ Few [doctors] have told me that…she is very kind*.*”* [41 years old, 12 years living with HIV]

In general, not many women shared whether their providers knew about their sex work occupation. One 37-year-old woman who has been living with HIV for 7 years explained how that information would not be relevant to disclose as *“those are her own personal things*.*”* This woman also indicated not discussing with her provider how alcohol consumption could affect her treatment, despite this issue coming up in her interview. In contrast, one woman did suggest that her provider knows about her sex work occupation, she explained:

*“There was something that the doctor told me*,*…he told me because my viral load was twenty*, *because it was undetected and now it is whatever [alluding to a high viral load]*, *he told me ‘you are not taking the medications correctly*.*’ I say*, *‘no doctor*, *it is not that I am not taking it correctly*, *what is happening is something*, *that is*, *I am working in the street sometimes if the medicine [I have to take it] at 9 in the morning and at that moment I’m not in the right place to take it or I take it an hour later or two hours later or when I remember or something like that’ he said ‘no*, *you set an alarm or see how to organize yourself*.*’”* [39 years old, 19 years living with HIV]

In disclosing information on sex work occupation, this participant had the opportunity to better explained the contextual barriers to medication adherence and engaged with her provider about possible strategies that could work for her.

While most women focused on the positive aspects of the relationship they have with their providers, some women also described instances when their providers did not treat them well and felt disrespected. On one occasion, a 21-year-old woman that was diagnosed at birth with HIV described the negative relationship she had with her provider, which led to her transferring to another clinic for her HIV care. She recalled that her provider was not forthcoming in explaining the rationale for making ART regimen changes and “spoke badly” to her. The same participant recommended that to improve the HIV care experiences of FSWs, care should be provided with dignity:

*“[My recommendation is for doctors to] be more educated*, *that is why there is education*, *that is why they went to school to [learn] that a patient is spoken to with dignity…Not like animals*, *it’s like*, *for example*, *I’m a cashier*, *don’t tell me that while I’m a cashier*, *I’m going to speak badly to the customer*, *because then I’m the one who loses*.*”* [21 years old, diagnosed with HIV at birth]

#### Continuity of care

Among women who reported receiving care by different providers, there was a sense of frustration in not having the same provider delivering HIV care during each visit. Seeing a different provider inhibited women’s ability to build trusting relationships with them. One woman explained her frustration with recent changes at her clinic:

*“Now they send me another doctor*, *every time there are different doctors…Every time a different face*. *No*, *[I don’t like that]…[Now] every time you come*, *there is a different face*, *sometimes they treat you well*, *sometimes they treat you badly*.*”* [32 years old, 7 years living with HIV]

Another woman explained the importance of continuity of care stating:

*“Yes*, *I would like to have only one doctor…Sure*, *because as I said*, *there are things that you only share when you only have one doctor like when I was with [my previous doctor]*, *there is trust and that whatever happened to you*, *you could say it*, *but when you see one today*, *one tomorrow*, *one the other day*, *another one on another day*, *and another one you just don’t have that same trust*.*”* [41 years old, 12 years living with HIV]

For some participants, lack of continuity of care presented a barrier to obtaining appropriate HIV care. In particular, participants would often deal with clinic confusion about basic information, such as what ART regimen they were on, which generated mistrust in the clinic environment. One participant explained:

*“Because there are doctors*, *look on one of these days they could not even find my chart*, *one [doctor] did not even see my chart*, *how are they going to give me the [ART] medicine if they did not find my chart*, *unless they know by heart what I was taking…*, *and I said ‘but come on here*, *a [medical] chart cannot disappear the day that you know [the patient] has an appointment’*, *[they say] there was a change [of staff]*.*”* [39 years old, 14 years living with HIV]

Interestingly, on another occasion a different participant described an experience wherein the lack of continuity of care also led to asserting more control over her own health and treatment.

The same distrust in the health care environment that stemmed from lack of continuity of care, was met with increase agency about how to navigate the health care environment. This participant described the following experience:

*“They give me a ticket [to be seen by doctor]*. *There I wait for my turn with the ticket*, *then they call me*, *we get stuck in a little room*, *sometimes [the doctor] forgets [my ART regimen] if I don’t walk with the medicine container [in hand]*, *there are no medicines for me every time I go*, *I always carry my container in my purse because well there are doctors*, *there are different doctors*, *so [I do this] so they don’t give me [the wrong treatment] and kill me…because sometimes the doctor says if I don’t walk with the container*, *he won’t know what medicine I take*. *Because they don’t search the chart much*. *Or if you tell them the name of the drug*, *they will find it for you*. *Many times I have been left without medication because I forgot the container*.*”* [32 years old, 7 years living with HIV]

#### Effective communication: Exploring participants’ treatment literacy

Most women were able to refer to their viral load results as an indicator for assessing their health and current HIV status. Furthermore, women often attributed ART adherence as the main factor that contributed to achieving a “good” viral load. As explained by one participant:

*“Being virally suppressed means that the virus is suppressed just like the word says*, *that is*, *that the medicine keeps the virus locked in*, *so to speak*, *boxed*, *it is suppressed*, *and the medicine does not allow it to leave [from] there to destroy my CD4*, *and destroy my body*.” [41 years old, 3 years living with HIV]

When probed about whether women knew if they had an undetectable or detectable viral load, or when expanding on their most recent viral load and CD4 test results some women had difficulty being able to fully express their understanding and address treatment literacy questions. These participants noted that they rely on their providers to let them know if something was not okay during their appointments.

*“No [I don’t know what viral suppression or what undetectable is]*, *and look that I’m with [my doctor] today*. *She gave [my analysis results] to me today that I had done*, *but since I can’t read it…They give them to me*, *I give it to the doctor and when the doctor sees them she says to me ‘Ah*! *No*, *it is all good’”* [37 years old, 12 years living with HIV]

For some women, having an understanding of their treatment and how viral load and CD4 count were affected by treatment allowed them to better advocate about their health and address provider concerns regarding their own level of adherence to medication. While discussing her current viral load status, one woman explained her concerns given her own level of adherence:

*“[Doctors] tell me I am not taking [my medication]… I take the medication*, *then I tell [my doctor] yes*, *yes*, *that he has to know that [the medications] are not doing anything to me because I am sure that I am taking it*. *No*, *no [he has not sent me to get tests done]*. *[Apart from viral load] he hasn’t told me to do anything*, *so that’s what I want him to do for me [to get more tests done]*. *Because one time that happened when I didn’t take [my medication] as I told you*, *it happened that I wasn’t doing anything and they changed it to another [medication because the viral load went up]… [But this time]*, *it didn’t come out [the viral load results were high] and like I was doing so well*, *it didn’t come out as I was [doing well]*.*”* [43 years old, 18 years living with HIV]

Indeed, effective communication and having a broader comprehension about HIV treatment allowed women to better navigate the patient-provider dynamics during clinical appointments.

## Discussion

Findings from this qualitative study highlight the importance of both structural and relational aspects of HIV care for FSWs. Furthermore, our results recognize the important contribution that a person’s agency has on the HIV care experience.

As past studies have demonstrated, dissatisfaction with the clinic environment could lead to disengagement in HIV care and poor HIV outcomes [24,32,44–46]; as such, the clinic environment and procedures, or structural aspects of quality of HIV care, are of great importance. First, as detailed in our results, it is important to consider clinics’ ability to keep a sufficient stock of ART when working with all people living with HIV (PLHIV), including multiply stigmatized groups experiencing socio-structural barriers like FSWs. The shortage of ART stock created a significant obstruction in the HIV care experience of FSWs. This shortage resulted in multiple clinic visits to collect ART refills, and most importantly, also led to changes, sometimes multiple, in the ART regimen for women who had otherwise been satisfied with their treatment due to its effectiveness and their familiarity with it. Furthermore, our findings underscore how imperative it is for the clinics to address ART stock shortage as this medication shortage impacted FSWs’ ability to control their own treatment adherence, supporting evidence linking ART shortages with poor treatment adherence [24]. Given that FSWs represent a key population in global efforts to address the HIV epidemic by reducing HIV incidence and risks for onward HIV transmission [47], this is a structural barrier that must be addressed to support efforts for treatment adherence and curve the HIV epidemic in this and similar contexts.

In addition, we identified that cost is a significant barrier or salient concern for assessing quality HIV care for FSWs. The costs associated with engaging in HIV care services creates a significant barrier for FSWs’ ability to sustain engagement with care services and could lead to the disillusionment of engaging in formal HIV care. Transportation costs, in particular, were often cited as the main deterrent to access HIV care and be satisfied with the current HIV care services. Participants also expressed that high frequency clinic visitation demands, often linked to medication pick-up, created a financial burden for themselves and their families as it implied incurring transportation costs, and possible costs related to clinic fees and food consumption. These associated HIV care costs inhibited FSWs from full satisfaction of HIV care services, with FSWs supporting the need for programs to provide assistance in the form of monetary contributions or vouchers for groceries to alleviate the financial strain. Similar studies among more generalized populations of PLHIV have also highlighted the need to address structural barriers, specifically transportation challenges, as evidence suggests these barriers obstruct PLHIV’s ability to engage and be retained in HIV treatment and care services [48,49]. Future initiatives should consider effective strategies to reduce the cost associated with HIV care, including exploring options for the integration of differentiated models of care for HIV treatment [50,51]. A recent study in South Africa found that compared to clinic-based ART, home delivery and monitoring of ART was not only highly acceptable among unemployed and financially constrained individuals but improved ART adherence thus increasing viral suppression [51]. Similar strategies for delivering and monitoring ART should be explored among key population groups like FSWs in the DR, as it would ultimately reduce HIV related costs by reducing the frequent clinic visitation schedules, in turn optimizing adherence and improving viral suppression. Furthermore, long-acting, injectable ART, which offers a dosing schedule of every 8 weeks, also presents another opportunity to address the high clinic visitation schedules and costs associated with HIV care among FSWs in the DR. A recent study exploring clinical trial participants experience switching from daily oral ART to long-acting ART found that this new treatment modality afforded them the ability to live more “normal” lives without the emotional burden of needing to go to a clinical care facility every month [52].

Another key component to the delivery of quality HIV care services relates to the relational aspects of care. We found that FSWs often focused on the interpersonal aspects of care and the personal connections they have established with their providers as testaments to the delivery of quality HIV care. The patient-provider relationship proved to play a significant role in how FSWs navigated their own treatment and care. Experiencing poor treatment by providers -such as being treated carelessly, without respect or dignity- was noted as being a barrier to accessing quality HIV care and led to the disruption of FSWs’ current HIV care services, as women would often seek to terminate their current care and request a referral to other clinic locations. Women also reported an appreciation for healthcare providers that share clear and complete information as it relates to their HIV treatment and care. Unsurprisingly, FSWs recommendation to improve the quality of HIV treatment and care services also included a call for respectful treatment by healthcare providers. Studies have found that discrimination or being treated with contempt or disrespect by clinical care providers could hinder engagement and retention in care [53,54]; as such, delivering care with dignity forms a central aspect of how we assess the quality of care being rendered to patients. Sensitivity trainings of all clinical care providers could aid in promoting more compassionate clinic environments and foster positive and more trusting patient-provider relationships. Furthermore, in line with FSWs’ focus on the relational aspects of care, there was a call by participants to promote continuity of care in the HIV clinic environment. Our findings suggest FSWs desire to build more trusting relationships with their healthcare providers, and being exposed to different providers during clinic appointments thwarts their ability to form connections and engage in more open dialogue.

The last component that was highlighted by our study is FSWs’ agency or their capacity to have control over their own HIV treatment and care experiences. Results from our study indicated that FSWs were able to exert their own agency- via immersion in understanding their treatment and the biological implications of their treatment- to better advocate and demand for quality HIV care services. As FSWs gained a deeper understanding of their treatment and the effect of treatment on their own health, they were able to understand the impact of treatment adherence on their ability to achieve viral suppression or an undetectable viral load. With this information, FSWs were able to better discuss treatment expectations, barriers to adherence, or the need to change to other treatments. We found that it was the understanding of the effects of treatment adherence on viral load that generated patient-led discussion on the possibility of treatment resistance. Furthermore, despite women having a strong desire for continuity of care, our results also highlight how FSWs exert their own power and agency over their treatment when placed under a care environment wherein they are unable to trust their healthcare providers due to inability to rely on the consistent delivery of care by the same provider during each appointment. As such, though FSWs view provider discontinuity as an impediment to obtaining good quality care, it could also inadvertently promote greater agency among women finding themselves in this situation.

Given how the HIV care process is affected by these relational aspects of care, which are further exacerbated by intersectional stigma and discrimination related to HIV and sex work, greater emphasis should be placed on the integration of culturally appropriate and comprehensive peer education, navigation and support programs for FSWs in HIV comprehensive care centers in the DR. These programs would ensure that despite challenges in establishing continuity of care due to structural clinic constraints, FSWs are exposed to and are able make linkages with known care workers in clinical spaces. Furthermore, the use of peer educators and navigators would allow for a more comprehensive approach to increase treatment literacy and improve clinic-level treatment and satisfaction by addressing staff efficiencies while providing greater opportunities for more dignified, inclusive and equitable care for this population [55].

This study is not without limitations. FSWs who are part of the parent study were recruited through the *Abriendo Puertas* intervention, a multi-level intervention designed to promote prevention and care for FSWs living with HIV in the Dominican Republic [8,9], and as such, women in our study likely represent a group of FSWs with greater established engagement with HIV care. Nevertheless, our study aimed to fully capture the experiences surrounding their engagement with HIV care, which expanded to understanding factors that have led to this engagement. Furthermore, given that women in our sample are part of a longitudinal cohort study, they have had greater exposure to working with investigators and responding to sensitive questions regarding their sex work occupation and HIV care experiences. In turn, our sample may reflect women with a higher level of comfort discussing sex work and their care experiences. Though this may limit the generalizability of our results, it also presents an opportunity for more thorough exploration given the trust and rapport built with participants in our study. Lastly, given that our research question was not the central focus of the parent longitudinal qualitative cohort study, our data on quality of HIV care experience is limited to broader, more general questions regarding FSWs’ experiences in HIV care. This may have hindered our ability to explore quality of care among FSWs living with HIV with greater circumspection. However, the use of two rounds of qualitative data per participant allowed for a richer immersion into the HIV care experience of women. As such, we believe the findings of our study present an important contribution to understand the dynamics that contribute to the care experiences of FSWs living with HIV.

Findings from this study have important implications for how programmatic efforts address and assess the quality of HIV care among FSWs. These findings provide an opportunity to understand the most pressing aspects that shape the quality of the HIV care experienced by FSWs, and thus affect FSWs’ pathway through the HIV care continuum. Our study highlights the importance of addressing the physical and social clinic environment by ensuring the availability of ART and addressing financial barriers, promoting positive and effective patient-provider dynamics, and facilitating opportunities for education and comprehension about treatment. Fostering treatment literacy created a pathway for promoting agency among FSWs leading to their self-advocacy and ability to make demands for better treatment and care services.

## Supporting information

S1 Checklist Qualitative research COREQ checklist. (PDF)

S1 Questionnaire PLOS’ questionnaire on “Inclusivity in global research”. (DOCX)

S1 Interview guides Participant In-depth Interview Guides. (PDF)
