# Supplementary material for: Exploring the dynamics of the quality of HIV care experienced by female sex workers living in the Dominican Republic
Source: PLOS Glob Public Health. 2023 Apr 28;3(4):e0001479. doi: 10.1371/journal.pgph.0001479 (PMC10146439; doi:10.1371/journal.pgph.0001479)
Supplement: S1 Checklist — (PDF) [file pgph.0001479.s001.pdf]

Qualitative Research COREQ Checklist

No. Item	Guide questions/description	Reported on Page
**Domain 1: Research team and reflexivity**		
Personal Characteristics		
1. Interviewer/ facilitator	Which author/s conducted the interview or focus group?	Authors' contributions
2. Credentials	What were the researcher's credentials? e.g., PhD, MD	Page 6 (Study participants and data collection)
3. Occupation	What was their occupation at the time of the study?	Page 6 (Study participants and data collection)
4. Gender	Was the researcher male or female?	Page 6 (Study participants and data collection)
5. Experience and training	What experience or training did the researcher have?	Page 6 (Study participants and data collection)
Relationship with participants		
6. Relationship established	Was a relationship established prior to study commencement?	Study participants and data collection
7. Participant knowledge of the interviewer	What did the participants know about the researcher? e.g., personal goals, reasons for doing the research	Page 9 (Ethical considerations)
8. Interviewer characteristics	What characteristics were reported about the interviewer/facilitator? e.g., Bias, assumptions, reasons, and interests in the research topic	Page 6 (Study participants and data collection)
**Domain 2: Study design**		
Theoretical framework		
9. Methodological orientation and Theory	What methodological orientation was stated to underpin the study? e.g., grounded theory, discourse analysis, ethnography, phenomenology, content analysis	Page 4 (Theoretical framework for understanding quality of HIV care)
Participant selection		
10. Sampling	How were participants selected? e.g., purposive, convenience, consecutive, snowball	Page 5 (Study design) and Pages 5 and 6 (Study participants and data collection)
11. Method of approach	How were participants approached? e.g., face-to-face, telephone, mail, email	Page 5 (Study design)

Qualitative Research COREQ Checklist

No. Item	Guide questions/description	Reported on Page
12. Sample size	How many participants were in the study?	Pages 5 and 6 (Study participants and data collection)
13. Non-participation	How many people refused to participate or dropped out? Reasons?	N/A
Setting		
14. Setting of data collection	Where was the data collected? e.g., home, clinic, workplace	Page 6 (Study participants and data collection)
15. Presence of non-participants	Was anyone else present besides the participants and researchers?	Page 6 (Study participants and data collection)
16. Description of sample	What are the important characteristics of the sample? e.g., demographic data, date	Page 9 (Results)
Data collection		
17. Interview guide	Were questions, prompts, guides provided by the authors? Was it pilot tested?	Pages 6 and 7 (Study participants and data collection)
18. Repeat interviews	Were repeat interviews carried out? If yes, how many?	Page 6 (Study participants and data collection)
19. Audio/visual recording	Did the research use audio or visual recording to collect the data?	Page 6 (Study participants and data collection)
20. Field notes	Were field notes made during and/or after the interview or focus group?	N/A
21. Duration	What was the duration of the interviews or focus group?	Page 7 (Study participants and data collection)
22. Data saturation	Was data saturation discussed?	Page 8 (Data management and analysis)
23. Transcripts returned	Were transcripts returned to participants for comment and/or correction?	N/A
**Domain 3: Analysis and findings**		
Data analysis		
24. Number of data coders	How many data coders coded the data?	Page 7 (Data management and analysis)

Qualitative Research COREQ Checklist

No. Item	Guide questions/description	Reported on Page
25. Description of the coding tree	Did authors provide a description of the coding tree?	N/A
26. Derivation of themes	Were themes identified in advance or derived from the data?	Page 8 (Data management and analysis)
27. Software	What software, if applicable, was used to manage the data?	Page 8 (Data management and analysis)
28. Participant checking	Did participants provide feedback on the findings?	N/A
Reporting		
29. Quotations presented	Were participant quotations presented to illustrate the themes/findings? Was each quotation identified? e.g., Participant Number	Results
30. Data and findings consistent	Was there consistency between the data presented and the findings?	Results and Discussion
31. Clarity of major themes	Were major themes clearly presented in the findings?	Results
32. Clarity of minor themes	Is there a description of diverse cases or discussion of minor themes?	N/A
