# Supplementary material for: Exploring the dynamics of the quality of HIV care experienced by female sex workers living in the Dominican Republic
Source: PLOS Glob Public Health. 2023 Apr 28;3(4):e0001479. doi: 10.1371/journal.pgph.0001479 (PMC10146439; doi:10.1371/journal.pgph.0001479)
Supplement: S1 Interview guides — (PDF) [file pgph.0001479.s003.pdf]

Stigma, Social Cohesion and HIV

Guide for the first interview with the qualitative cohort

Date: _________________________ Interviewer: _______________________________

Interview Location: _________________________PID: _______________________ __

Introduction

Thank you for taking the time to speak with me today. We appreciate your participation and are happy to be able to talk with you today about your ideas and experiences. We are interested in hearing about how you take care of your health and things that help you stay healthy and things that sometimes get in the way. In particular we are interested in talking about how you manage your HIV status and your ability to access HIV care services and take your medications.

1. I'd like to start by getting to know you a little better. Can you tell me about who you live with and where you live?

- How do you feel in this living arrangement?
- How do you get along with the people you live with?

2. Who are the most important people in your life at this time? This can be people in your family, community and beyond. Let's make a list of these people that are most important to you.

(Probe below for each person on the list)

- What is your relationship to this person?
- How do they support you?
- How do you support them?

3. Can you tell me about your current work/employment situation?

- What are all of the different things you to do make money?
- What kind of environment are you working in?
- How do you meet/connect with clients?

4. Are you connected to a group or organization of other sex workers? Tell me about that…

- What types of activities do you do with this group?
- What types of support do you receive?
- What challenges are there with this group?

5. Now I would like to hear about your health. In general, how do you feel in terms of your health?

- How healthy do you currently feel? Tell me more about how you feel…

6. What things help you to feel and stay healthy? Tell me more…

7. What things get in the way of you feeling and staying healthy?

8. Now I would like to hear about your experience with HIV. Can you tell me about when you found out that you were living with HIV? Tell me about when you were diagnosed.

- How did you feel?
- How did you react to your diagnosis?

9. Who have you told about your diagnosis?

10. How do you feel talking to people about HIV?

11. How was your health when you were diagnosed?

- How did you feel at this time?

12. Tell me about the process of starting to receive HIV care and treatment after your diagnosis.

- How long did it take after you were diagnosed for you to have your first appointment with a doctor (or other health professional) and start treatment?
- What do you remember feeling during this time about needing treatment for the rest of your life?

13. Coming back to the present/today, how you are doing managing your HIV care and treatment?

14. Where do you go to get your medication/your ART?

- How often do you pick up your ART pills?
- Are you always the person who picks up your ART pills?
- Does anyone else pick up your ART pills?
- Have you ever shared ART with someone else (given or received pills from someone else)?
  - Who do you share ART with?
  - How often do you share your ART with this person/these people?

14. How do you know how you are doing in terms of managing your HIV?

- What helps you know how you are doing with your HIV?
- What information does your doctor (or other health professional) give you to help you know how you are doing with your HIV management?
- Does your HIV doctor know about your work as a sex worker? Tell me more about that…

15. Are you familiar with the concept of viral suppression or having an undetectable viral load? What does that mean to you?

- How do you know if you are suppressed or undetectable?

16. Do you know if you are currently virally suppressed (or have an undetectable viral load)?

For those who say they are undetectable:

17. How long have you been undetectable?

18. How have you been able to become virally suppressed? What helped you to become undetectable?

19. What things have helped you to be undetectable? Tell me more about those…

20. Have you ever not been undetectable Tell me about what was going on when you were not undetectable.
- When you were diagnosed, do you remember if your viral load was undetectable?
- Have you ever had a period since your diagnosis when you went from being undetectable to detectable? Tell me about this…

21. What makes it hard to be undetectable?

22. If you have to identify the most important thing to help you be undetectable, what would it be?

23. How have the people we talked about earlier helped you to be undetectable?

24. Was there ever a time when you stopped taking your HIV medicines? Tell me about that time…
- What was going on at this time?
- What affected your daily routine with your medications?

25. How common is it to stop taking medication among other female sex workers living with HIV that you know?
- What makes it hard for other female sex workers living with HIV to take their medication?

26. How does the experience with medication compare among women living with HIV who are sex workers and women who are not sex workers?

27. In general, do you think being a sex worker affects your viral load? How so?
- How do people who know you are a sex worker treat you?
- How do people in the clinic treat you?

For who say they are not undetectable:

28. Have you ever been undetectable? When was that?

29. Tell me from your perspective why you aren't currently undetectable?

30. What keeps you from being undetectable?
- What makes it hard or complicated for you to be undetectable?

31. Have you ever stopped taking them for a period of time? Tell me about this time…

32. Have you ever not been very consistent in your daily medication routine? Tell me more about this….

33. How common is it to stop taking medication among other sex workers you know living with HIV?

34. And how common is it to stop taking medication among other women living with HIV who are not sex workers?

35. In general, do you think being a sex worker affects your viral load? How so?
- How do people who know you are a sex worker treat you?
- How do people in the clinic treat you?

36. What would it take for you to become virally suppressed in the next few months?

For all participants:

37. Now, if there was the option of an HIV treatment that you would receive as an injection (in the backside) once a month or once every two months at a clinic, and it worked just as well as the daily pill to control your HIV (keep you suppressed)…how would you feel about that injectable HIV treatment option?
- ¿What do you think of an injectable treatment compared to a daily pill?

38. What are the advantages of an injectable form of treatment from your view?

39. What are the disadvantages of an injectable form of treatment from your view?

40. What would other sex workers think about an injectable treatment? Tell me more about your thoughts on this…

We have come to the end of my questions. Thank you for sharing your experiences with me. Before we end, do you have any questions for me? Or, is there anything else you would like to share? Thank you again for your time and insights.

Stigma, Social Cohesion and HIV

Guide for the second interview with the qualitative cohort

Date: _________________________ Interviewer: _______________________________

Interview Location: _________________________PID: _______________________ __

Introduction:

Thank you for taking the time to speak with me today. We appreciate your participation and are happy to be able to talk with you again about your ideas and experiences. We are interested in continuing the conversation that we started last year and hearing more about how you take care of your health and things that help you stay healthy and things that sometimes get in the way.

I. Social context

1.1 To begin, I would like to know how has this last year been for you? Tell me about how your life has been since the last time we talked…

- What is the most important thing that happened in your life this past year?
- During the last year, how have you felt about yourself?

1.2 How is your family?

- And your kids? Tell me about how your kids have been...

1.3 Do you have a steady partner right now? Tell me about this partner and your relationship…

1.4 Has anything changed in terms of your living arrangements since the last time we spoke? Tell me about who you are living with at the present time.

- How do you get along with the people you live with?

1.5 Now I would like to talk about the people in your life, as we did last year. Take a minute and think about who are the most important people in your life and we can make a list of these people. They can be family, neighbors, partners, friends, people you work with etc…. *(use social network inventory form to make a list of the names and then use probes)*

- What is your relationship with ______________________?
- What does ____________ mean to you?
- Does _________know you are living with HIV?
- What do you talk about with____________________?
- How does __________support you? (probe: financially, emotionally, etc…)
- How do you support___________? (probe: financially, emotionally etc….)

Is there anyone else who is important to you that you did not mention?

1.6 At the present time, do you have friends/acquaintances that you can count on unconditionally? Tell me about these friends/acquaintances… (*use probes above*)

1.7 How has your social network changed after your diagnosis with HIV?
- Are there people you were connected to before that you are no longer in contact with? Can you give me an example?
- Have you made new connections with people after your diagnosis?

1.8 Now I would like to ask about the community/neighborhood that you live in. How do you feel living in this community/neighborhood?
- What is your relationship like with your neighbors?
- How do your neighbors treat you? Tell me more about this…

1.9 Since the last time we talked, have you been judged or mistreated by anyone in your family or your community/neighborhood? Tell me about what happened….
- Who judged or mistreated you?
- What did they say?
- Why do you think they did this?
- Any other example of mistreatment from this past year?

1.10 What did you do when you were mistreated?

1.11 What do you do to avoid mistreatment?

II. Work and economic situation

2.1 Tell me about your work/employment situation during the last year.
- Tell me everything you do to make money.
- On a typical day, what are all of the things you do to make money?

2.2 Are you currently doing any type of sex work?
- In what kind of environment are you working? Or How are you meeting clients?
- Tell me about your work schedule on a typical day.

2.3 How do you feel about doing sex work?

2.4 Have you changed your way of doing sex work since you were diagnosed with HIV? Tell me more….
- For example, have you changed where you work? Tell me about this…
- Have you changed the number of clients you are seeing? Tell me more…
- Have you made any changes to the way you meet and communicate with clients? Explain this to me…

2.5 What is your relationship with other women you work with?
- How do you treat each other?

2.6 What have you told them about your health?

- How did they react?

2.7 Overall, how is your economic situation?

- Has your economic situation affected your HIV care and treatment?
- Tell me, how has it affected?

2.8 Since the last time we talked, how has your nutrition been?

- At any time have you not had enough food? Tell me about this…

2.9 How does it affect you when you don't have enough food?

- In the last year, have you changed your medication routine due to lack of food? Tell me about this…

III. Social cohesion and participation

3.1 Are you involved in any group or organization of sex workers? Can you tell me more about this….

- What group(s) are you involved with?
- When did you get involved?

3.2 How has your involvement been with this group during the last year?

- What have you done with this group?
- What activities have you participated in?
- What has aided or limited your participation in activities with this group?
- Has anything changed with this group in the last year? Tell me about this…

3.3 What does this group mean to you?

- Do you trust the members of this group? Tell me more…
- How do you feel when you are meeting or in an activity with this group?
- How does this group support you (financially, emotionally, etc…)

3.4 How do the women in this group treat you? Tell me more about this…

- Do you feel like part of a community with the other women in this group? Can you tell me more about this…

3.5 What do the women in this group know about your HIV status?

- How did they react?

IV. Health and Wellbeing (mindfulness)

4.1 Now I would like to talk to you a little more about your health. Overall, how have you felt since the last time we spoke?

- How have you felt physically?
- How have you felt emotionally?
- Have you had any health situations this last year? Tell me about this…

4.2 What helps you to feel well? Can you give me an example of something you do to stay healthy?

4.3 What things have made it hard for you to feel good and stay healthy?

4.4 Have you ever felt stressed during the last year? Tell me what caused this stress.
- How did this stress affect you?
- How did you feel?
- Tell me, how did this stress affect your healthcare routines and daily activities?

4.5 What did you do to feel less stress?
- Where did you learn to do this?
- Have you received any counseling or psychological care this last year? Tell me more about this…
- Have you ever participated in an activity or workshop to reduce stress? Tell me about this activity….

V. HIV care and treatment

5.1 How are you doing with your HIV care? Tell me how thing have been going with your care this past year.

5.2 Has anything about your care changed? (probe: Clinic? Doctor? Meds?)

5.3 Tell me about your appointments at the clinic. Walk me through everything you do during an appointment from the time you arrive to the time you leave the clinic.
- How often do you go for HIV care?

5.4 Since the last time we spoke, have you stopped going to the clinic for a while or have you missed an appointment? Can you tell me more about this…?

5.5 How do you feel in the clinic where you get your HIV care?
- How are you treated?

5.6 During the last year, have you ever been mistreated at the clinic? Tell me about this…

5.7 What things have helped you with your HIV care during this past year? Tell me more about these things…
- What else has helped?

5.8 Who is the person who has helped you the most with your HIV care during the last year? When I say help, I mean economically, logistically, emotionally, spiritually etc…. Can you give me an example of how this person has helped you?

5.9 In what ways do the people you mentioned to me at the beginning of the interview help you with your HIV care? *(refer back to their social network to probe)*

5.10 What things have made it difficult for you to get HIV care? Can you give me some examples?

5.11 How does sex work affect your HIV care and treatment?
- How does it affect your attendance at appointments?
- How does it affect your adherence to medication?
- What else?

5.12 Last year we talked about viral suppression. Do you know what your viral load is currently? In other words, do you know if you are detectable or undetectable?
- What does this mean to you?
- What do you think could have influenced you being detectable o undetectable?

5.13 Now I would like to hear about your medication. Tell me about the medications you take for HIV.
- During the last year, have you had any changes to your medications?
- How many times and for what reasons did you have these changes?
- Previously, how many times had you changed your medications? For what reasons?

5.14 How do you feel about taking these medications every day?

5.15 - How do you remember to take your medication? Tell me everything you do and who helps you…

5.16 During the last year, have you ever stopped taking your medication? Tell me more about this……
- What was going on at this time?
- What affected your daily medication routine?

5.17 What would help to improve your overall experience with HIV care and treatment?
- What kinds of programs do you think would be most helpful to you?

5.18 What is the hardest part for you about living with HIV?

VI. Mobility

6.1 Where do you consider "home?" Is it in Santo Domingo or outside of Santo Domingo? Tell me about this…

6.2 Think about all of the different places you go in Santo Domingo and outside of Santo Domingo in the last year. Please tell me all of the different places you went where you spent at least one night. (*Make a list of all places and probe for each place*)
- **When** do you typically go? Is there a particular season or time of year?

- **Why** do you go there? If for work, what type of work do you do there? Do you exchange sex for money when you travel there?

6.3 Tell me about a typical/recent trip you've made outside of Santo Domingo in the last year.

- **Where** did you go?
- **How long** were you gone?
- **Preparations**: What kind of arrangements did you have to make before you traveled? [e.g. places to stay, transportation, food, time off work, care for children, telling friends/family, HIV care appt etc.]
- **Trip**: How did you get there?
  - Did you travel with anyone? Who? Did you stop anywhere else along the way? Describe.
  - Did you have any inconveniences or difficulties? Any specific dangers/fears?
- **Why** did you go there?
- Describe any violence you experienced before, during, and/or after you got to your destination.
- Did you exchange sex for money when you traveled there? [Probe on direct and/or indirect sex-related activities.]
  - How are conditions there different compared to your regular work environment in Santo Domingo? [Probe on circumstances surrounding sex work and type of clientele]
  - Did you consume any alcohol or drugs? Tell me about this…
- **Needs**: What were your greatest needs while you were traveling?
- **Care-seeking/adherence**:
  - Did you access HIV services while you were outside of Santo Domingo? Did you have any problems accessing these services?
  - What about taking your medications for HIV while you were outside of Santo Domingo? Tell me about this…
- **Social relationships**: Did you have any friends/family where you went?
  - Who did you see while you were traveling?
  - Did you feel like you had support there? What kinds of support (e.g., emotional, sense of community, resources, organizations)? From whom?
  - Did you contact anyone while you were traveling? Who? How often?
- **Cell phone use**: How did you communicate while you were traveling?
  - Do you use a cell phone when you travel? Could you receive text messages or phone calls while traveling?
  - How does your cell phone use change when you are traveling compared to when you are in Santo Domingo?
  - If they don't have their own cell phone: Why do you not have a cell phone of your own?

6.4 Would you want programs to be able to reach you when you are traveling?

- In your opinion, how could they best reach you when you are outside Santo Domingo?
- Have you ever called a call/help center [Note: to adapt language to incorporate locally relevant language] while traveling? What services did you receive? What other services could a call/help center offer that you think would be beneficial for you when traveling?
- If someone were to call or text you while you were traveling, what resources or services in particular could they offer that you think would be beneficial for you when traveling?

Thank you so much for taking the time to speak with me today. Do you have any questions?
Is there anything else you would like to share?
